# Application Values of Six Scoring Systems in the Prognosis of Stroke Patients

**DOI:** 10.3389/fneur.2019.01416

**Published:** 2020-01-30

**Authors:** Qun-Xi Li, Xiao-Jing Zhao, Hai-Yan Fan, Xiang-Nan Li, Da-Li Wang, Xiu-Jie Wang, Jiang Zhang, Rui-Ying Chen, Li Zhang

**Affiliations:** ^1^Department of Neurosurgery, Affiliated Hospital of North China University of Science and Technology, Tangshan, China; ^2^Department of Neurology, Affiliated Hospital of North China University of Science and Technology, Tangshan, China

**Keywords:** APACHE, CSS, NIHSS, ADL, GCS, evaluate, prognosis, stroke

## Abstract

**Objective:** The present study aimed to evaluate the prognostic value of Acute Physiology and Chronic Health Evaluation (APACHE; II and III), Chinese Stroke Scale (CSS), National Institutes of Health Stroke Score (NIHSS), activities of daily living (ADL) (Barthel index, BI), and Glasgow Coma Scale (GCS) scores for stroke patients.

**Methods:** A total of 352 stroke patients were evaluated using APACHE II, APACHE III, CSS, NIHSS, ADL, and GCS scores within 24 h after admission. And these patients were consecutive admissions to the hospital. The endpoint was in-hospital death. The scores of these scales were compared between the survival group and death group, and the receiver operating characteristic (ROC) curves were drawn. The ability of each scoring system to predict the prognosis of patients was evaluated using the area under the ROC curve, and the areas under the curves (AUCs) of these six scales were compared.

**Results:** The AUCs of the APACHE II, APACHE III, CSS, NIHSS, ADL, and GCS scores were 0.882, 0.867, 0.832, 0.859, 0.838, and 0.819, respectively.

**Conclusion:** APACHE II, APACHE III, CSS, NIHSS, ADL, and GCS scores have good predictive values in the prognosis of stroke patients. APACHE II is superior among the other five scales.

## Introduction

Stroke is a common and prevalent disease which has the characteristics of high incidence, high mortality rate, high disability rate, high recurrence rate and many complications. Stroke, along with heart disease and malignant tumor, are the three leading causes of death in most countries, which bring heavy economic, and mental burden to families and the society. To date, there are many methods to assess stroke at home and abroad, but the most haven't been widely adopted. Moreover, in order to prove the value of strengthening medical treatment, evaluate the condition of patients with different diseases and compare the therapeutic effects of different treatment schemes, a unified assessment criterion is needed. Therefore, it is necessary to comprehensively evaluate some of the major stroke scoring methods presently used around the world. The investigators intended to compare Acute Physiology and Chronic Health Evaluation (APACHE) II and III (1, 2), Chinese Stroke Scale (CSS) (3), National Institutes of Health Stroke Score (NIHSS) (4), activities of daily living (ADL) (Barthel Index, BI) (5), and Glasgow Coma Scale (GCS) score (6). The same stroke patient was comprehensively assessed to compare the application values of these six scoring systems in the prognosis of stroke, providing reference for the correct selection of stroke scale.

## Information and Methods

### Patient Selection

A total of 352 patients with acute stroke, who were admitted in the Department of Neurology and Department of Neurosurgery, Affiliated Hospital of North China University of Science and Technology from January 2015 to December 2016 were enrolled in the present study. All selected patients were admitted to the hospital within 3 days after onset. The diagnoses of all patients were in accordance with the diagnostic criteria for cerebrovascular disease developed by the Fourth Academic Conference of National Cerebral Vascular Disease in 1995, and confirmed by head computed tomography (CT) or magnetic resonance imaging (MRI). The following patients were excluded from the study: patients with transient ischemic attacks; patients with mild conditions who had only sensory symptoms or a muscle strength not lower than grade IV; patients with serious dysfunction of heart, liver, kidney, and other organ which could become the main reason of influencing the prognosis of patients; patients who have a history of stroke, and could not take self-care of themselves; patients who did not comply or could not complete the tests; patients with hematological and neoplastic cerebral hemorrhage; patients with atherosclerotic cerebral infarction or cerebral embolism.

### Evaluation Method

Stroke patients who met the inclusion criteria were evaluated using the following six scoring scales within 24 h after admission: APACHE II, APACHE III, CSS, NIHSS, ADL, and GCS scores. All receiver operating characteristic (ROC) curves of APACHE II, APACHE III, CSS, NIHSS, ADL, and GCS scores to predict the prognosis of patients were drawn, and the areas under the ROC curve (AUCs) were calculated. The area under the ROC curve of the two scoring systems was compared by the method come up with by Hanley and McNeil (7). The calculation formula and the concrete steps were as follows:

$$\begin{array}{l} ①\text{Z = }\left({\text{A}}_{1}-{\text{A}}_{2}\right)\text{/}\;{\left({\text{SE}}_{1}^{2}{\text{+ SE}}_{2}^{2}-{\text{2rSE}}_{\text{1}}{\text{SE}}_{\text{2}}\right)}^{\text{0}.\text{5}}\\ \;②{\text{r}}^{\text{a}}\text{=}\left({\text{r}}_{\text{N}}{\text{+ r}}_{\text{A}}\right)\text{/2}\\ \;③{\text{A}}^{\text{a}}\text{=}\left({\text{A}}_{1}{\text{+ A}}_{\text{2}}\right)\text{/2} \end{array}$$

r_N_ was the related coefficient between the two scores of survival patients. r_A_ was the related coefficient between the two scores of the dead patients. r was the related coefficient of the area under the ROC curve of the two scores. A_1_ was the area under ROC curve of score 1. A_2_ was the area under ROC curve of score 2. SE_1_ (Standard Error of Area 1) was the standard error of area under ROC curve of score 1. SE_2_ (Standard Error of Area 2) was the standard error of area under ROC curve of score 2.

Using the values of r^a^ and A^a^, the value of r was obtained by looking up tables. The above results were brought into the formula to obtain the *Z*-value of the normal distribution statistics. *Z*-score was obtained by looking up tables. When *Z* ≥ 1.96 and *P* < 0.05, it could be concluded that the area difference between the two scoring ROC curves was significant.

### Statistical Analysis

All data were analyzed using SPSS 13.0 statistical software. Measurement data were compared using *t*-test. All tests were two-sided tests. *P* < 0.05 was considered statistically significant.

## Results

### Gender, Consciousness State, Lesion Sites, and Lesion Types in the Death Group and Survival Group (Table 1)

A total of 352 patients were enrolled into the present study. Among these patients, 196 patients were male and 156 patients were female. Furthermore, among these patients, 26 patients had lacunar infarction, 220 patients had cerebral infarction, 80 patients had cerebral hemorrhage, and 26 patients had mixed cerebrovascular disease. In addition, bilateral lesions were found in 112 patients, left lesions were found in 110 patients, right lesions were found in 106 patients, and brainstem lesions were found in 24 patients. Moreover, when they were admitted to hospital, 242 patients were conscious and awake, 38 patients were sleepy, 14 patients were lethargic, 22 patients were in mild coma, 16 patients were in moderate coma, and 20 patients were in deep coma.

**Table 1:** Consciousness state composition of the survival group and death group [*n* (%)].

**Treatment outcome**		**Survival group**	**Death group**
Gender	Male	128 (65.3)	104 (66.7)
	Female	68 (34.7)	52 (33.3)
Consciousness state	Conscious and awake	200 (82.6)	42 (17.4)
	Sleepy	22 (57.9)	16 (42.1)
	Lethargic	4 (28.65)	10 (71.4)
	Shallow coma	4 (18.18)	18 (81.8)
	Moderate coma	0 (0)	16 (100)
	Deep coma	2 (8)	18 (90)
Lesion site	Brainstem lesions	2 (8.3)	22 (91.7)
	Bilateral lesions	84 (75)	28 (9)
	Right lesions	66 (62.3)	40 (37.7)
	Left lesions	80 (72.7)	30 (27.2)
Lesion type	Lacunar infarction	24 (92.3)	2 (7.7)
	Cerebral infarction	150 (68.2)	70 (31.8)
	Cerebral hemorrhage	42 (52.5)	38 (47.5)
	Mixed cerebrovascular disease	16 (61.5)	10 (38.5)

### Comparison of Scores Between the Survival and Death Groups

Table 2 shows that on the first day of admission, the differences in APACHE II, APACHE III, CSS, NIHSS, GCS, and ADL scores of patients with acute stroke between the survival group and death group were statistically significant, the APACHE II, APACHE III, CSS, and NIHSS scores were significantly higher in the death group than in the survival group, and the GCS and ADL scores were significantly lower in the death group than in the survival group.

**Table 2:** Comparison of scores in hospitalized patients with acute stroke between the survival group and death group.

**Scoring methods**	**Survival group (*n* = 232)**	**Death group (*n* = 120)**	**AUC**	**SE**	***t***	***P***	**95% CI**	
							**Lower**	**Upper**
APACHE II	6.65 ± 4.11	15.86 ± 6.51	0.882	0.023	−13.913	0.0001	0.837	0.927
APACHE III	21.91 ± 10.54	49.95 ± 23.27	0.867	0.024	−12.352	0.0001	0.821	0.913
CSS	14.89 ± 10.26	30.54 ± 11.53	0.832	0.027	−12.424	0.0001	0.779	0.885
NIHSS	7.26 ± 6.04	19.33 ± 8.35	0.859	0.024	−13.823	0.0001	0.812	0.907
GCS	14.18 ± 2.49	9.36 ± 4.32	0.819	0.030	10.935	0.0001	0.761	0.877
ADL	50.64 ± 29.51	13.96 ± 22.57	0.838	0.026	12.513	0.0001	0.787	0.890

### Comparison of the Validity of Scoring Systems in Predicting the In-Hospital Mortality of Patients With Acute Stroke

**Figure 1:**
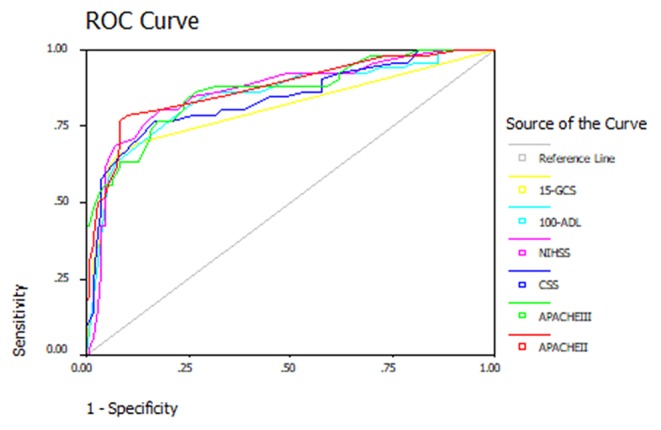
The ROC curve of six scores of ACVD patients on the 1 day of admission.

**Figure 2:**
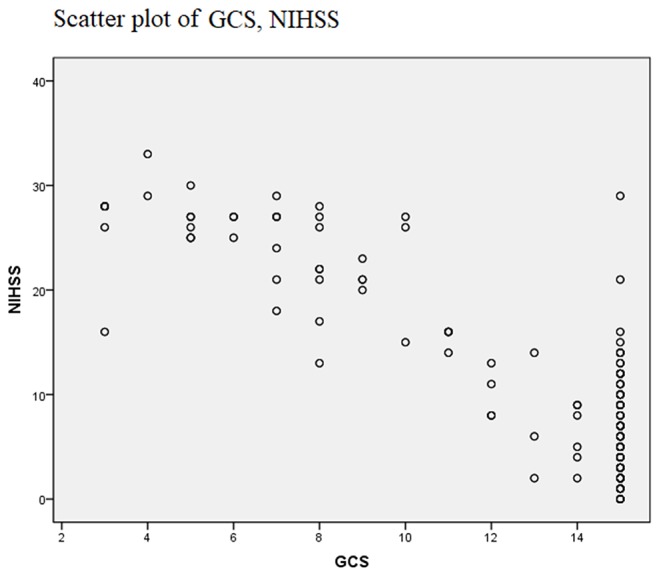
Scatter plot of baseline distributions of GCS, NIHSS.

Evaluating the validity of a scoring system in judging the prognosis of a disease can be confirmed through the ROC curve. First, with the actual in-hospital death or survival of patients with acute stroke as the gold standard for the prognosis of patients, the sensitivity and specificity of these scoring systems at each point were calculated. High APACHE II, APACHE III, CSS, and NIHSS scores reflect more severe stroke impairment while lower ADL and GCS scores reflect more severe stroke impairment. Therefore, ADL and GCS scores were used to construct the ROC curves, in order to conform to the principle that the higher the score, the more serious the disease became. The ROC curves of these scoring systems at admission were drawn (Figure 1). Baseline distributions of GCS, NIHSS were provided as scatter plots (Figure 2), the AUCs were calculated, and the differences between the AUC of these scoring systems and area under the baseline (0.5) were compared (Table 2). Comparisons of AUC were conducted between any pair of six scoring systems (Table 3). Based on the ROC curves, the cut-off values and the predictive power in predicting prognosis of stroke patients according to 6 scoring systems were listed in Table 4.

**Table 3:** Comparisons of AUC between two of six scoring systems of the prognosis of stroke patients.

**Comparison group**	**r_**N**_**	**r_**A**_**	**(r_N_+r_A_)/2**	**(A_1_+A_2_)/2**	**r**	***Z*-value**
APACHEII and GCS	0.634	0.744	0.689	0.851	0.62	3.6309^*^
APACHE and CSS	0.292	0.587	0.439	0.857	0.38	1.7834
APACHEII and NIHSS	0.468	0.658	0.563	0.871	0.48	0.9591
APACHEII and vADL	0.271	0.53	0.4005	0.86	0.33	1.5457
APACHEII and APACHEIII	0.781	0.716	0.7485	0.8745	0.67	0.7848
APACHEIII and GCS	0.556	0.526	0.541	0.843	0.48	1.7134
APACHEIII and CSS	0.261	0.468	0.3645	0.8495	0.3	1.1563
APACHEIII and NIHSS	0.406	0.597	0.5015	0.863	0.43	0.3121
APACHEIII and ADL	0.172	0.472	0.322	0.8525	0.26	0.9522
CSS and GCS	0.522	0.708	0.615	0.8255	0.09	0.3375
CSS and NIHSS	0.874	0.909	0.8915	0.8455	0.87	−2.0266^*^
CSS and ADL	0.878	0.755	0.8165	0.835	0.78	−0.3408
NIHSS and GCS	0.737	0.808	0.7725	0.839	0.73	1.9407
NIHSS and ADL	0.733	0.776	0.7545	0.8485	0.71	1.0978
GCS and ADL	0.393	0.582	0.4875	0.8285	0.12	−5.098^*^

*When Z-value was < 1.96, P-value was more than 0.05,which meant there was no significant difference in area under ROC curve between the two scoring methods*.

* *Represented that there was significant differences*.

**Table 4:** Sensitivity and specificity of six scores in predicting prognosis of stroke patients.

**Score systems**	**Score**	**Sensitivity**	**Specificity**
APACHE II	8	0.808	0.815
	9	0.788	0.898
	10^*^	0.769	0.917
	11	0.731	0.917
	12	0.917	0.917
APACHE III	24	0.885	0.667
	25	0.885	0.685
	26^*^	0.865	0.731
	27	0.827	0.759
	28	0.808	0.759
CSS	24	0.769	0.787
	25	0.769	0.796
	26^*^	0.769	0.833
	27	0.712	0.870
	28	0.692	0.889
NIHSS	12	0.750	0.861
	13	0.712	0.880
	14^*^	0.692	0.926
	15	0.673	0.935
	16	0.615	0.954
100-ADL	65	0.865	0.574
	70	0.865	0.685
	75	0.846	0.713
	80^*^	0.808	0.759
	85	0.635	0.926
15-GCS	2	0.692	0.880
	3	0.673	0.898
	4^*^	0.654	0.926
	5	0.615	0.944
	6	0.558	0.944
	7	0.500	0.944
	8	0.385	0.963

* *The cut-off value of the six score systems*.

*APACHE II, acute physiology and chronic health evaluation II; APACHE III, acute physiology and chronic health evaluation III; CSS, Chinese Stroke Scale; NIHSS, National Institutes of Health Stroke Score; ADL, activities of daily living; and GCS, Glasgow Coma Scale*.

Table 2 revealed that all these scoring systems had predictive value for the in-hospital mortality of patients with acute stroke, and had good validity. The ROC curves for the APACHE II, APACHE III, CSS, NIHSS, ADL, and GCS scores on the first day of admission were drawn, and the AUCs for the APACHE II, APACHE III, CSS, NIHSS, ADL, and GCS scores were 0.882, 0.867, 0.832, 0.859, 0.838, and 0.819, respectively. These results revealed that the APACHE II, APACHE III, CSS, NIHSS, ADL, and GCS scores were valuable in predicting the in-hospital mortality of patients with acute stroke. From Table 3, it could be concluded the AUC of APACHE II and GCS, CSS, and NIHSS, and between GCS and ADL had statistical significance, while AUC of other items in Table 3 had no statistical significance. Table 4 revealed that the score of Apache II was 10 points, Apache III was 26 points, CSS was 26 points, NIHSS was 14 points, 100-ADL was 80 points (ADL was 20 points) and 15-GCS was 4 points (GCS was 11 points) with the largest Jordan index. Therefore, the cutoff of APACHE II, APACHE III, CSS, NIHSS, ADL, and GCS scores were 10, 26, 26, 14, 20, and 11 points, respectively.

## Discussion

Objective and accurate evaluation of the severity of the disease in critical patients is of great significance for the judgment of the disease. The purpose of the application of the scoring method for critical disease is to quantitatively evaluate the severity of disease, and predict the risk of disease or death of patients (8, 10, 11). This is based on patient indicators, such as acute physiological changes, anatomical changes, and even chronic disease lesion factors, and the severity of the disease can be objectively quantified by assigning values, weighting, logical reasoning, and complex mathematical operations (12). This quantitative evaluation result is very important for doctors to understand the condition and dynamically observe the changes in disease. For stroke patients, stroke scale is the only approach to solve this problem (13). Quantifying the prognosis of stroke patients can more accurately evaluate the severity of the disease, and predict the risk of death, providing a scientific basis for evaluating the condition of critical patients. According to these scores, one can judge the condition of the disease, and more reliably predict the mortality rate of a patient population, providing an objective basis for doctors, family members, and the society to make medical decisions, help correctly formulate the treatment plan and explain the condition to the family members. Furthermore, it can assess the selective operation of monitoring patients in the intensive care unit (ICU) (6), and is helpful in promoting the in-depth study of stroke. The evaluation of the neurological deficit and prognosis of stroke has been carried out for half a century, but there is presently no uniform standard.

The general pathogenesis of stroke is sudden vascular rupture or vascular obstruction in the brain, which causes interruption of the channels through which blood flows into the brain, induces ischemia and hypoxia in brain tissues, and subsequently damages brain tissues (14). A related study revealed that (15) stroke has become the first cause of death in China, and also the primary cause of disability in adults. A study revealed that the prognostic factors in stroke patients generally include health status, systemic reactions, and the severity and nature of intracranial lesions (16).

The APACHE scoring system is a commonly used scoring system, which includes health status, age and physiological scores. The APACHE III scoring system was expanded and developed based on the APACHE II scoring system, and these two have good consistency in death probability. The APACHE III scoring system fully takes the severity of visceral dysfunction, age and the patient's own functional status into account, which is commonly used to predict the severity of all types of patients (17, 18).

NIHSS is the most widely used scale for evaluating the severity of stroke in the world, and this has been mainly used to evaluate the severity of neurological deficit, curative effect and prognosis (19–22). At present, the NIHSS scoring system is commonly used to assess the severity of stroke patients and predict mortality in clinic, and helps physicians take active intervention measures to improve the prognosis of critical patients (23). The NIHSS scoring system only scores 11 indicators of neurological function, and neglects factors that affect the prognosis, such as age and chronic health status. Therefore, this has some limitations in judging the severity and prognosis of the disease (24).

The CSS score has been revised and modified based on the Scandinavian Stroke Scale (SSS) by Qingtang Chen *et al*., which was adopted at the Second National Conference on Cerebrovascular Diseases, and its projects were revised again at the Fourth National Conference on Cerebrovascular Diseases in 1995. Jing Su in China revealed that (25) the internal consistency of NIHSS in stroke patients is relatively high, which has predictive validity for the prognosis of stroke, and has the best structural validity in some patients with anterior circulation. Zirong Tao also considered that the CSS score has better reliability, validity and sensitivity in stroke patients (9). Furthermore, a study revealed that (26) the AUC of the CSS score for the prognosis of patients with acute cerebral infarction was 0.796.

The GCS score is a coma scale first developed for patients with cerebral surgery in 1974 by Teasdale et al. (27). This has also been commonly used in stroke patients, and was revised in 1976 (28). Clinicians usually use GCS to evaluate the level of consciousness state of stroke patients, and its assessment of nervous system functional defects focuses on uncommon symptoms of acute stroke patients, such as difficulty in eye closure, decerebration, and decortication symptoms, but these do not include aphasia and sports defect classification. For patients with stroke, GCS often overestimates the degree of neurological deficit. Therefore, it is not suitable for stroke patients without impaired consciousness, but for patients with aphasia.

BI was developed in the mid 1950s, and was designed and applied in clinic by Horence Mahoney and Dorothy Barthel in the United States. This was called the Maryland disability index at that time, was officially called the Barthel index (BI) in literatures in the mid 1960s, and is being used at present. It is simple in terms of evaluation, and has high credibility and sensitivity. Furthermore, it is one of the most widely used and most widely studied ADL evaluation methods, it can not only be used to assess the functional status before and after treatment, and can predict the outcomes of treatment, length of hospital stay and prognosis. Furthermore, it is a common method in rehabilitation medical institutions in the United States, and is also one of internationally recognized ADL evaluation methods (29). This index was first published by Dorothea Barthel and F Mahoney in 1965. It is an effective index to judge the recovery of limb function in stroke patients, and is a commonly used evaluation method in rehabilitation medicine. However, its prediction for stroke death has not been reported.

The APACHE II, APACHE III, CSS, NIHSS, ADL, and GCS scores are used to evaluate the condition of stroke patients. However, the comparison of predictive values among these six scoring methods in stroke patients has not been reported.

The present study revealed that the APACHE II, APACHE III, CSS, and NIHSS scores were significantly higher in the death group than in the survival group, and ADL and GCS scores were significantly lower in the death group than in the survival group. The differences in these six scoring systems between the survival group and death group were statistically significant (*P* < 0.001). These results reveal that all six scores can predict the prognosis of stroke patients, which is consistent with that reported in previous literatures. The present study also revealed that the AUCs for the APACHE II, APACHE III, CSS, NIHSS, ADL, and GCS scores were 0.882, 0.867, 0.832, 0.859, 0.838, and 0.819, respectively. It is general considered that the larger the area under the general recognition curve, the more authentic it is to predict the prognosis of patients. This suggests that all these six scores have good validity for the evaluation of the prognosis of stroke patients. Chen et al. considered that (30) the APACHE II score has important clinical significance in evaluating the prognosis of stroke patients. Bein et al. conducted a comparative study on the comparison between APACHE II and APACHE III in 150 patients in the ICU (31). The results revealed that both APACHE II and APACHE III have good prognostic values, and it was concluded that the area under the ROC curve for APACHE II was 0.847, and the area under the ROC curve for APACHE III was 0.899. A study in China confirmed that compared with the APACHE II scoring system, the APACHE III scoring system is more accurate in predicting death, and more reasonable and scientific in system design. Furthermore, the study also revealed that the APACHE III scoring system was more accurate in assessing the disease condition for patients with internal diseases (32). In addition, another study revealed that (33) for stroke patients, the AUC of APACHE III was 0.848, the sensitivity was 0.749, the specificity was 0.802, the standard error was 0.017, and the 95% confidence interval was 0.816–0.881. That study revealed that the application of the APACHE III scoring system could be applied for a more scientific and reasonable clinical evaluation of the condition and prognosis of acute stroke patients, providing a reliable basis for actively implementing intervention, effectively improving the prognosis of patients, and increasing the survival rate of patients. In addition, we evaluated the scores on the first day, the third day and the seventh day of admission. Through statistical analysis, we calculated the predictive value of various scores at different time points. The score and node with the largest predictive value were determined by comparing the area under ROC cur so as to predict the prognosis of the patients with acute stroke more accurately in the future. This is the rationale why 24 h was used as the reference timepoint.

Quantifying the prognosis of stroke patients can accurately evaluate the severity of the disease, predict the prognosis of the patients, help to make a correct treatment plan and explain the condition to the family members. It has been generally considered that the greater the AUC, the higher the validity for the prediction of the prognosis of patients. In clinical practice, the area under ROC curve of prediction index is usually between 1 and 0.5. By comparing the area under ROC curve, the clinician can intuitively know whether an index has reference value in disease prediction and can compare the prediction efficiency of each index by statistical method.

Therefore, the present study considered that the APACHE II and APACHE III scores in predicting the prognosis of patients with acute stroke should be emphasized.

However, our study still have some limitations. Firstly, the result of scoring systems may be influenced by subjective factors to a great extent. So our research results need to be verified by prospective research of large sample. Secondly, this study was still in the initial stage of research, and lacked comprehensive, systematic and large-scale clinical dynamic assessment, which was also the direction of our future research.

In summary, scale assessment has been widely applied in clinical and scientific research, which plays a great role in promoting the improvement of medical level. Each scale has its own application range, as well as its own advantages and disadvantages. No scale can completely measure and predict all types of strokes, and all changes in its indicators. Therefore, the accurate selection and application of the appropriate evaluation scale play an important role in judging the condition, making treatment decisions, and evaluating the treatment efficacy and prognosis of the disease. There is presently no ideal gold standard for assessing the prognosis of patients with acute stroke. The preliminary study conducted by the investigators revealed that the order of clinically recommended prognostic scoring methods for stroke was as follows: APACHE II, APACHE III, NIHSS, ADL, CSS, and GCS. APACHE II is preferentially recommended.

## Data Availability Statement

The datasets generated for this study are available on request to the corresponding author.

## Ethics Statement

This study was carried out in accordance with the recommendations of the declaration of Helsinki and the ethical committee of Affiliated Hospital of North China University of Science and Technology, with written informed consent from all subjects. All subjects gave written informed consent in accordance with the Declaration of Helsinki. The protocol was approved by the ethical committee of Affiliated Hospital of North China University of Science and Technology.

## Author Contributions

Q-XL been involved in drafting the work and revising it critically for important intellectual content. X-JZ given final approval of the version to be published. H-YF was involved in article revision. X-NL and X-JW made substantial contributions to the conception and design of the work. JZ, D-LW, R-YC, and LZ made substantial contributions to the acquisition, analysis, and interpretation of data for the work.

### Conflict of Interest

The authors declare that the research was conducted in the absence of any commercial or financial relationships that could be construed as a potential conflict of interest.
